# Intrinsic Breaking of Internal Solitary Waves in a Deep Lake

**DOI:** 10.1371/journal.pone.0041674

**Published:** 2012-07-23

**Authors:** Martina Preusse, Marek Stastna, Heinrich Freistühler, Frank Peeters

**Affiliations:** 1 Environmental Physics, Limnological Institute, University of Konstanz, Germany; 2 Department of Applied Mathematics, University of Waterloo, Ontario, Canada; 3 Department of Mathematics and Statistics, University of Konstanz, Germany; Philipps-University Marburg, Germany

## Abstract

Based on simulations with the Dubreil-Jacotin-Long (DJL) equation, the limiting amplitude and the breaking mechanisms of internal solitary waves of depression (ISWs) are predicted for different background stratifications. These theoretical predictions are compared to the amplitude and the stability of the leading internal solitary waves of more than 200 trains of ISWs observed in the centre of a sub-basin of Lake Constance. The comparison of the model results with the field observations indicates that the simulated limiting amplitude of the ISWs provides an excellent prediction of the critical wave height above which ISWs break in the field. Shear instabilities and convective instabilities are each responsible for about half of the predicted wave breaking events. The data suggest the presence of core-like structures within the convectively unstable waves, but fully developed and stable cores were not observed. The lack of stable trapped cores in the field can be explained by the results from dynamic simulations of ISWs with trapped cores which demonstrate that even slight disturbances of the background stratification cause trapped cores to become unstable.

## Introduction

The degeneration of basin-scale waves to ISWs and of the ISWs to turbulence by wave breaking is one of the main processes of energy transfer from large to small scales in the thermocline and deeper water regions of lakes [1], [2]. Due to the enhanced energy dissipation and mixing observed near the lake and ocean boundaries [3], [4], attention concerning wave breaking in the field has primarily been directed to ISWs interacting with sloping topography [5], [6], [7], [8]. It is however known that the stability of ISWs in undisturbed water is determined by the ISW amplitude [9], [10], [11], [12]. The amplitude of an ISW grows if the wave's energy increases (e.g. due to a decrease in total depth or energy gain from the ISW generation mechanism, e.g. a steepened large-scale seiche). If the wave amplitude exceeds its limiting amplitude the wave starts to break. Hence, ISWs can also break in the lake interior away from topographical features [13]. Two qualitatively different mechanisms are responsible for ISW breaking in deep water, breaking due to shear instabilities or breaking due to convective instabilities, which results in the formation of a trapped, or recirculating, core [11]. These breaking mechanisms have different ecological consequences, in lakes as well as in the ocean. A shear limited wave can be assumed to dissipate energy, thereby inducing local mixing in the thermocline. An ISW with a trapped core theoretically contributes less to mixing, but has the potential to transport particles enclosed in the core over large distances [14]. It is therefore important to classify the breaking mechanism of breaking waves in the field.

Limiting amplitudes required for the occurrence of both convective and shear instabilities have been studied numerically [9], [10], [11], [15] and in the laboratory [12]. The occurrence of the different breaking mechanisms strongly depends on the background stratification and the background shear current, if one is present [10], [16]. In the absence of a background current, waves with trapped cores can only be generated in stratifications without a mixed surface layer [11], [12]. Such strongly monotonic stratifications are common in mid-latitude lakes during the warming period. However, to the best of our knowledge, the numerical predictions have not been tested against field data, since statistical data about ISW properties are scarce.

Here we compare observed amplitudes and breaking events of the leading ISWs of over 200 measured wave trains with simulations based on the Dubreil-Jacotin-Long (DJL) equation. This numerical investigation provides a theory-based analysis of the observations presented recently by Preusse et al. [17] and demonstrates that ISW breaking in the field can be adequately predicted from the background stratification.

This study is organized as follows: after the description of the field experiments, the theoretical concepts and numerical methods, we compare the theoretical prediction of the limiting amplitude for the onset of ISW breaking and the corresponding breaking mechanism with field observations of ISW amplitudes and ISW breaking. A dynamic simulation of one of the convectively unstable ISWs observed in the data set demonstrates the sensitivity of the trapped core to upstream perturbations in the background stratification.

**Figure 1:**
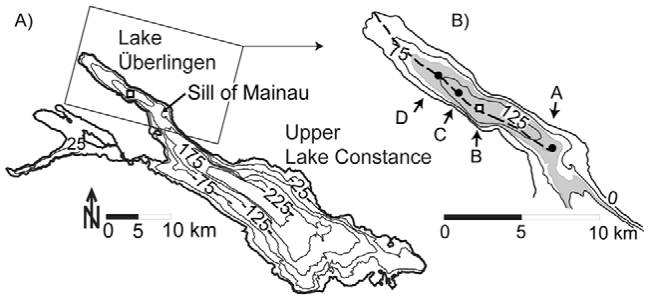
Experimental design. A) Bathymetry of Lake Constance and the location of the main study site (square), B) Zoom into Lake Überlingen with deployment stations from October 2010 (A–D), main study site B (square) and thalweg (broken line). The shaded area depicts depths ≥100 m.

**Figure 2:**
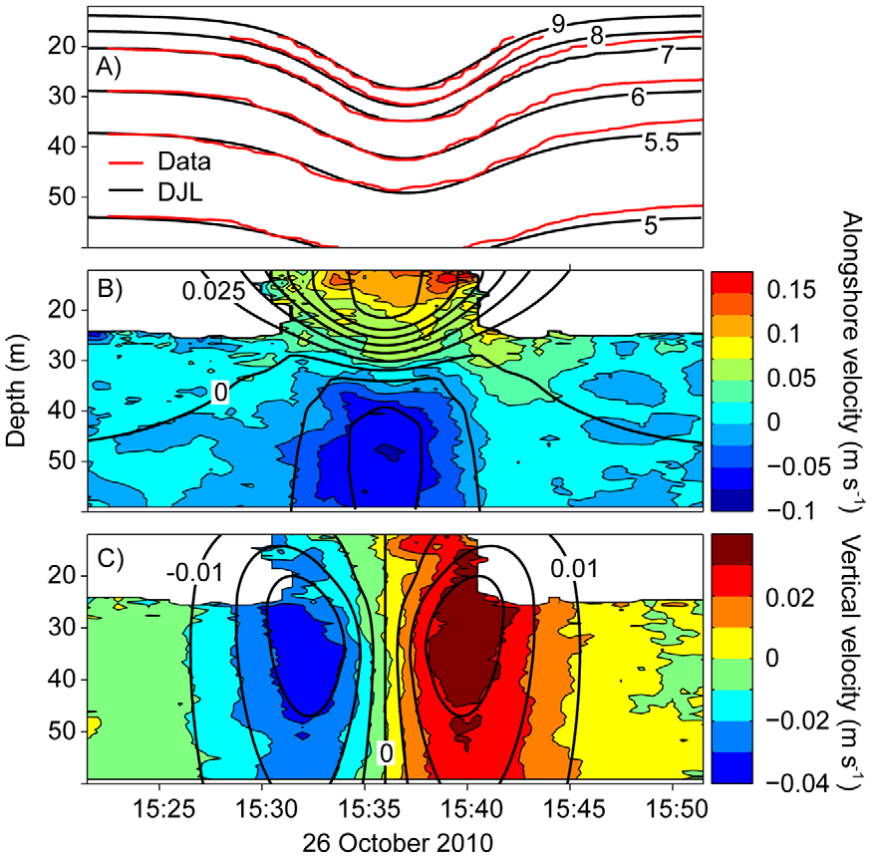
Simulation of a measured ISW. Fields of (A) temperature, (B) along-shore velocity (missing values are white) and (C) vertical velocity of a solitary-like wave as observed at station D (colours) and as simulated by the DJL equation (black thick lines).

**Figure 3:**
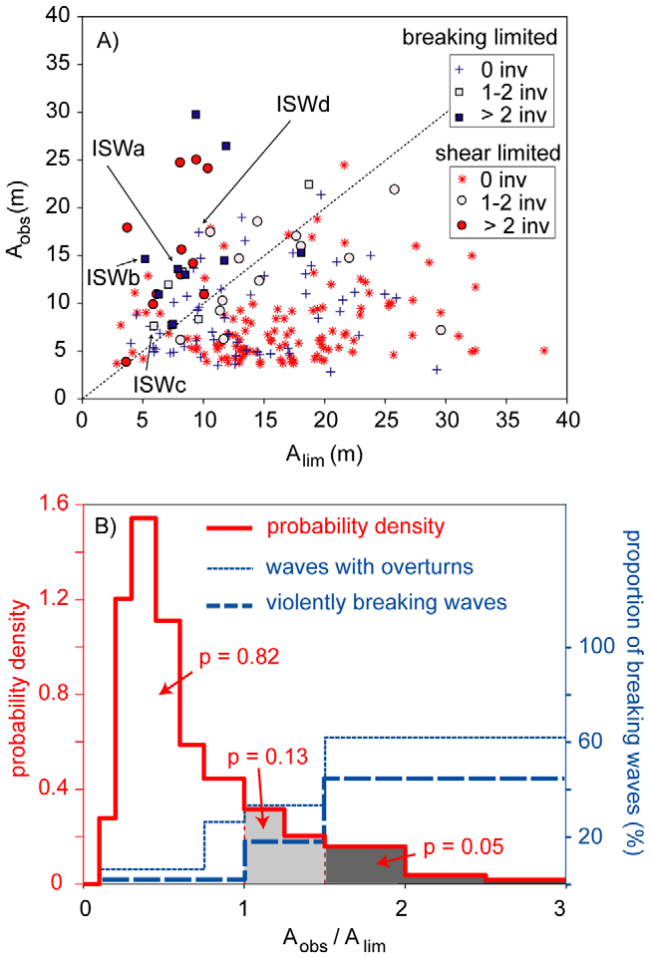
Correlation between the occurrence of ISWs with density inversions and ISWs with amplitudes exceeding their simulated amplitude limits *A_lim_*. (A) Scatter plot separating shear (red and circles) and breaking (blue and squares) limited ISWs and showing the correlation between *A_lim_* and *A_obs_.* The limit, above which breaking is expected, is indicated by the broken line. ISWs with density inversions are depicted as circles and squares, violently breaking waves are intensively coloured. ISWa – ISWd are shown in Fig. 5. (B) Probability to observe waves within ranges of *A_obs_/A_lim_* (red line, denoted as probability density, values for shaded areas are depicted) and probability to observe waves accompanied by density inversions (blue lines, denoted as proportion) within ranges of *A_obs_/A_lim_*.

**Figure 4:**
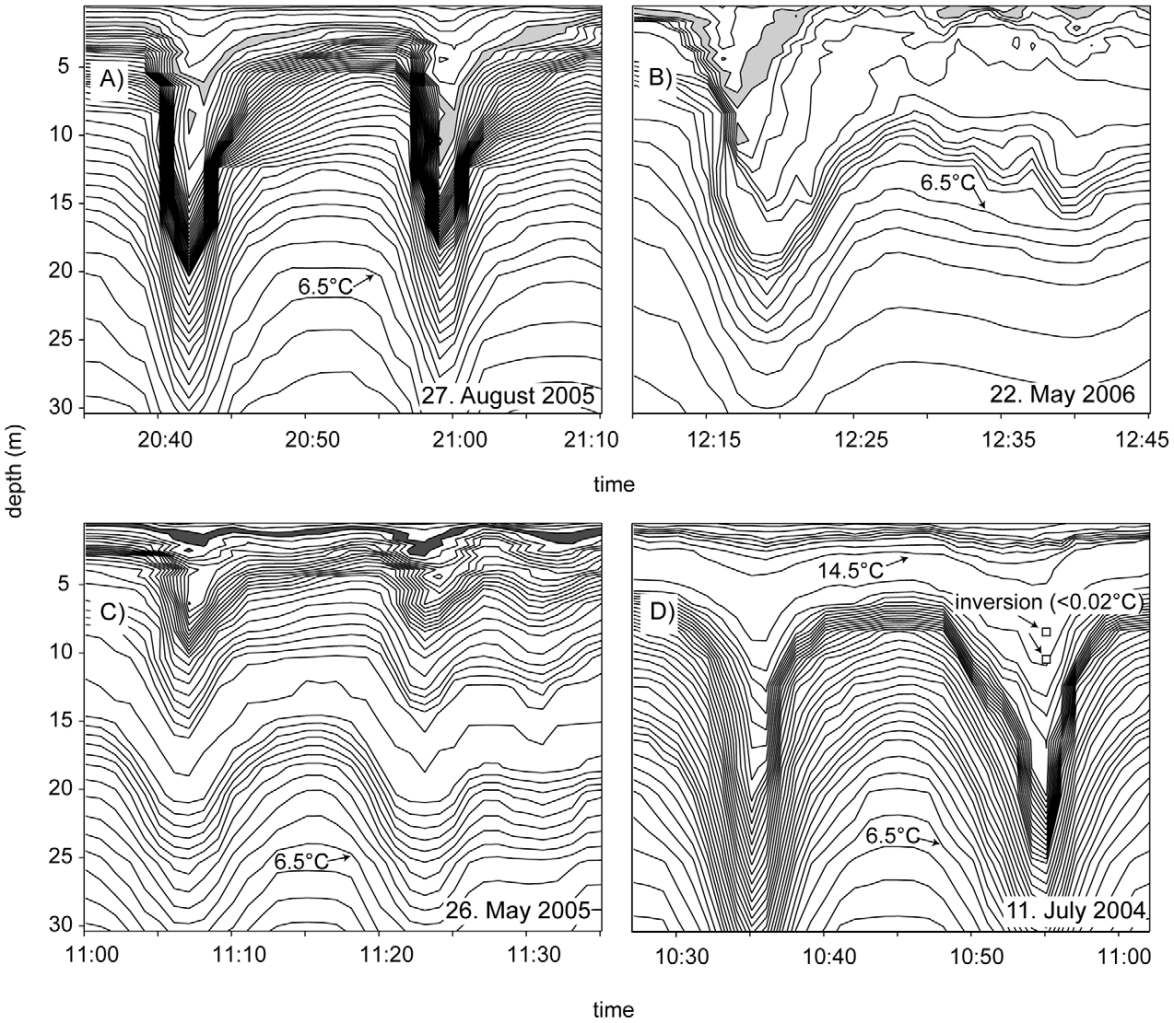
Measured breaking limited waves. Measured examples of (A) waves with trapped cores during autumn with a filled area between the contour-lines at 15.5°C and 15.75°C, (B) a wave with a perturbed trapped core during spring with a filled area between the contour-lines at 9.5°C and 9.75°C, (C) small waves with trapped cores during spring with a filled area between the contour-lines at 15°C and 15.25°C and (D) breaking limited waves without significant inversions during summer. The temperature difference spanned by the 2 m -inversion in the second wave is 0.014°C, slightly above the accuracy range of 0.01°C.

**Figure 5:**
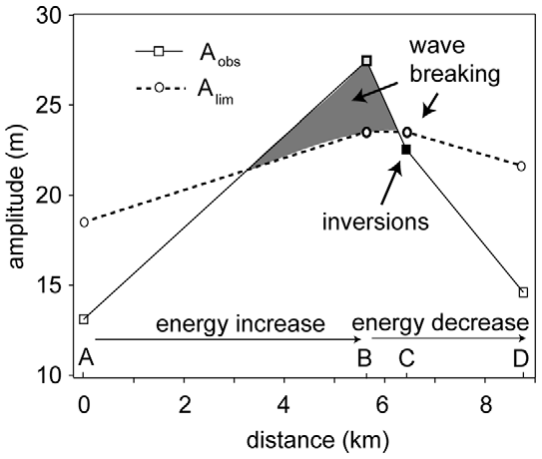
Increase and decrease of ISW amplitude with propagation distance of a shear limited wave observed on 26 October 2010. The grey area denotes simulated (*A_obs_/A_lim_* >1), the black square indicates observed (density inversions) wave breaking.

**Figure 6:**
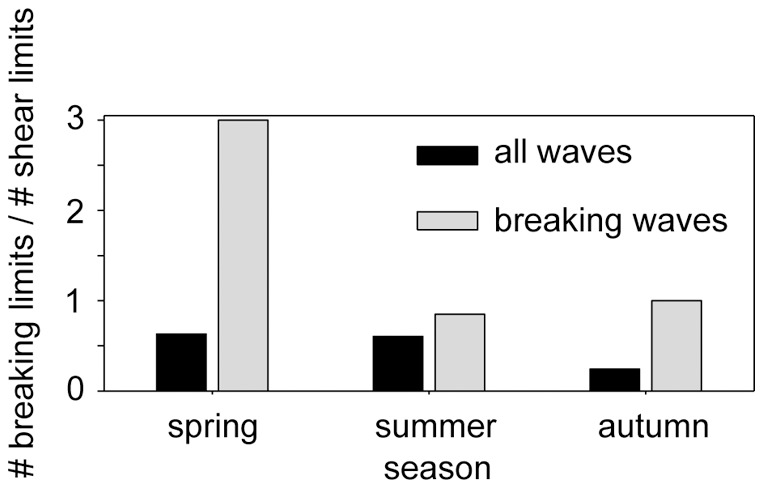
Ratio of the number of waves with amplitudes bounded by the breaking limit and waves with amplitudes bounded by shear limit depicted for different seasons (spring: April, May, summer: June – August, autumn: September, October). Black bars correspond to all waves, grey bars to waves simulated to break (*A_obs_/A_lim_* ≥1).

**Figure 7:**
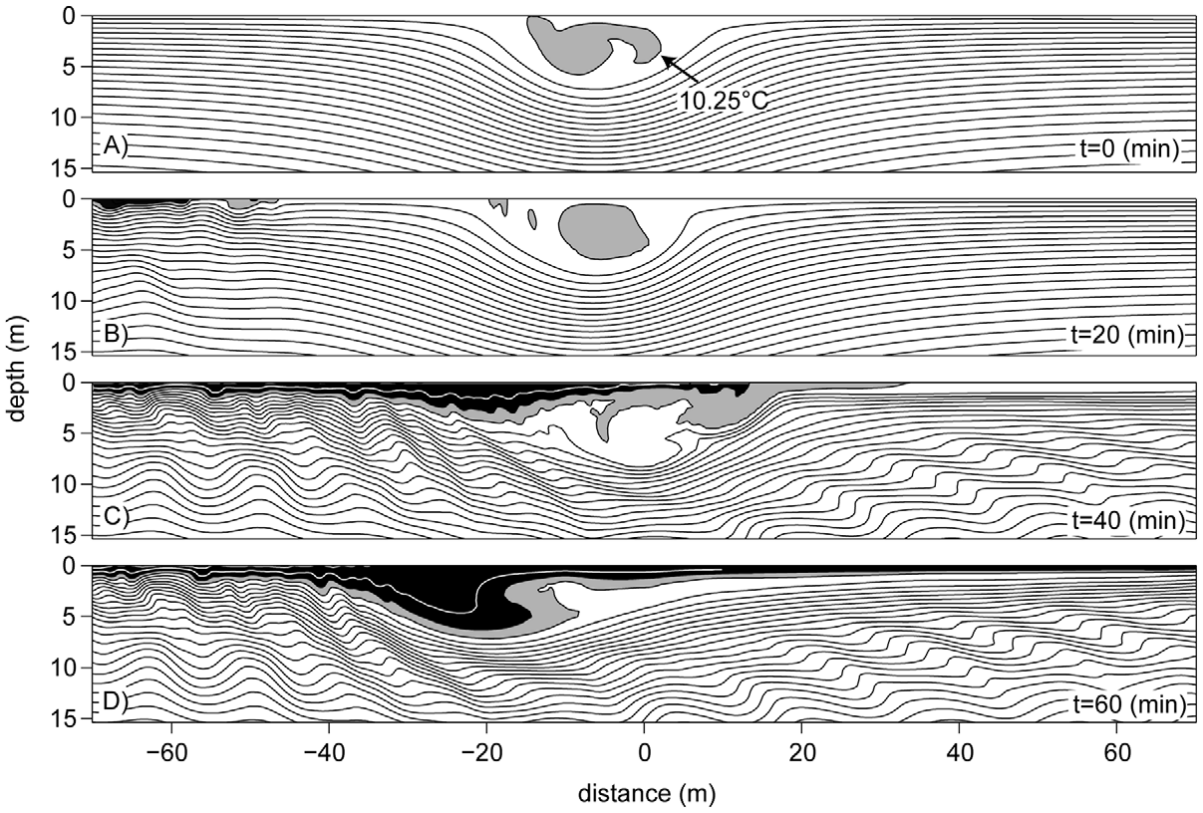
A simulated breaking limited wave encountering a perturbation. Dynamical simulations of (A) a wave with a quasi-steady core and (B–D) a perturbation of the wave in (A), 20, 40 and 60 min after a disturbance (a patch of light (black) fluid in the top 2.4 meters of the water column) was introduced upstream of the wave. Isotherms are given in 0.25°C intervals.

## Methods

ISWs in Lake Constance (Fig. 1A) propagate at the steepened front of the basin-scale seiche and are typically observed in the small subbasin Lake Überlingen [18]. At the deepest location of Lake Überlingen (Fig. 1B, station B) temperature profiles were collected almost continuously over 6 years (2004–2007 and 2009–2010) with a temporal resolution of one minute and an accuracy of 0.01°C (PME thermistor chain). The vertical spacing of the thermistors ranged from 10 m between 50 m and 130 m depth, 5 m between 20 m and 50 m depth, to a resolution of 2 m and finer between 20 m and 0.5 m. In order to obtain extrapolated density-profiles over the total water column for the simulations, the density gradient for the surface was chosen to be the same as we observed at 0.5 m below the surface. The data were analysed using an automatic detection method which allowed the identification of the occurrence of ISWs, the times of passage of the ISWs and an estimate of the amplitudes *A_obs_* of the ISWs [17]. The properties of the ISWs were estimated by fitting the empirical function
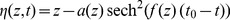
(1)


to measured isothermal depths of isotherms at 0.1°C intervals during the passage of an ISW. Thereby, *η(z, t)* is the vertical displacement of the isotherm with resting depth z at time t, where z lies between *z = *125 m (the surface) and *z = *0 m (near the lake bottom boundary layer). The fit was optimized for the parameters isothermal displacement *a(z)*, frequency *f(z)* and time of passage *t_0_* of the ISW trough. The amplitude *A_obs_* was derived as the maximum isothermal displacement, or max*(a)*.

In autumn 2010 we conducted a further field experiment during which temperature was measured with a time resolution of one second at three additional thermistor chains (RBR). The thermistor chains were deployed over one month in the center of Lake Überlingen (Fig. 1B). Lake depths ranged between 100 and 140 m; 100 m (station A), 140 m (station B), 135 m (station C) and 120 m (station D). Each chain was equipped with at least 13 thermistors spaced with a vertical distance less than 2 m in the thermocline. At the mooring at station D current velocity was measured with an ADCP in the upper 50 m of the water column with a time resolution of 30 s and a vertical resolution of 0.5 m.

## Results and Discussion

### Comparison to theory

In a frame of reference moving with the wave, an ISW is stationary. Such waves can be described by the DJL equation [10], [11], [19], given by
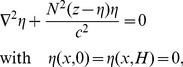
(2)where *η =  η(x, z)* is the vertical displacement of the isotherm passing through location *x* relative to its depth *z* at rest, z  =  H at the surface and H is the total water depth. The DJL theory makes no *a priori* assumption about wave amplitude (unlike weakly nonlinear theories such as the well-known Korteweg – de Vries equation), and is completely equivalent to the full set of stratified Euler equations. It is thus an ideal tool to study large amplitude waves.

Since the leading waves of an ISW train have the highest probability of being stationary when using a reference frame moving with the wave, we only simulated the leading ISW of each train. Assuming that the leading ISW is generated at or before the Sill of Mainau [20] or at the western end of Lake Überlingen after the reflection of the steepened front of the basin-scale seiche [18], the wave evolution distance from the location of generation to the longterm measuring station B in the center of Lake Überlingen is at least 5 km (Fig. 1A).

Solutions of the DJL equation as described in Stastna and Lamb [10] depend on the background stratification, background current and amplitude of the wave. Since we did not measure the current velocities at the longterm measuring station B, we applied the DJL equation (2) neglecting background currents, following the numerical scheme described in Stastna and Lamb [10]. To confirm the validity of this approach, (e.g. neglecting background currents) we simulated the leading ISW of a wave train observed on 26 October 2010 at station D (Fig. 2), a time during which current velocities were measured.

The simulations were based on the average of 60 temperature profiles measured within one hour before the passage of the leading ISW and on the measured amplitude *A_obs_* of the ISW. The predicted displacements of isotherms and current velocities associated with the ISW agree well with the observations (Fig. 2), suggesting that the wave was sufficiently stationary for the simulation with the steady state DJL equation. Moreover, the good agreement between observation and simulation suggests that background currents, for which velocities were smaller than 0.05 ms^−1^ upstream of the front generating the ISWs during the field experiment in autumn 2010, do not strongly influence the properties of ISWs in Lake Constance.

### Limiting amplitudes

The limiting amplitudes of the leading ISWs of the wave trains observed during the longterm experiment were estimated via the DJL equation by slowly increasing the amplitude of the simulated ISWs until the limiting amplitude is reached [11]. For the stratifications observed at the deep-water study site only two scenarios occur: First, the wave reaches an amplitude where the ISW induced current velocity equals the wave velocity and convective instabilities occur (often referred to as the breaking limit). Second, the variational algorithm used to compute the waves fails to converge to a solution. Waves close to the point at which the variational algorithm fails to converge have Richardson numbers at the wave crest that are near 0.25 [10]. For this reason we refer to this as the stability limit. Through a variety of ingenious techniques it is possible to augment the variational algorithm so that it can yield waves with much lower Richardson numbers [21], but these do not have significantly larger amplitudes and hence this approach was not pursued in our study. Waves for which the limiting amplitude *A_lim_* exceeds the observed amplitude *A_obs_* are predicted to break (Fig. 3A, upper triangle). Note that this procedure provides an extremely accurate threshold for the onset of convective instabilities, which occur if the ISW induced current velocity exceeds the phase velocity of the wave. The limiting amplitude for the onset of shear instabilities is however slightly underestimated, and the shear limit as derived here presents a lower threshold for the onset of shear instabilities. Of course the observed wave amplitude is altered by the inherent variability in the stratification, surface wave field and winds. As such, our basic hypothesis is that the limiting amplitude arrived at from a naïve application of the DJL equation should yield qualitatively accurate predictions of any breaking observed in the field.

This is a somewhat different point of view than that adopted in the recent literature on shear instability in ISWs. Recent numerical [9], [15], [21] and laboratory studies [12] showed that Richardson numbers substantially smaller than 0.25 are necessary for the occurrence of shear instabilities. However these studies either considered laboratory scales, which do not clearly correspond to the situation in our field measurements (a different stratification, in particular), or focused on instances of strong billow formation in a thermocline located well below the surface. None of the above-mentioned studies considered an active near surface layer (due to surface waves, for example).

Integration of the probability density of the occurrence of ratios *A_obs_*/*A_lim_* determined that 18% of 219 leading ISWs have ratios above one, i.e. that *A_obs_* is larger than the *A_lim_* (Fig. 3B, red line). This suggests that 1/5 of the leading ISWs observed in the open water of Lake Überlingen break or are at least close to breaking in the case of shear unstable waves. 5% of the leading ISW have amplitudes exceeding *A_lim_* by a factor of more than 1.5.

Temperature inversions occurring in association with ISWs within the depth range of the stratified thermocline imply density inversions and indicate instabilities and breaking of the ISWs. Examples of ISWs with temperature inversions are shown in Fig. 4. To distinguish real overturns from instrument noise we only considered temperature inversions that are characterized by a temperature difference of more than 0.02°C compared to the stable (sorted) temperature profile. This criterion is well above the accuracy range of the temperature measurements. Such well-resolved temperature inversions were observed in about 15% of the leading ISWs (Fig. 3A, filled markers). About 9% of the waves (60% of the waves with inversions) were violently breaking, i.e. were associated with more than two temperature inversions occurring at different times during the passage of the wave (Fig. 3B, thick blue line). About 60% of the ISWs exceeding their limiting amplitude by more than 1.5 were accompanied by inversions with temperature differences larger than 0.02°C (Fig. 3B, thin blue line). Most likely even more of these waves broke, but the rather coarse time (1 min) and depth (≥1 m) resolution of the temperature data only allows comparatively large overturns to be detected (Fig. 4D). The number of observations of ISWs accompanied by overturns increases with *A_obs_/A_lim_*. This indicates that either the amount, or size, of overturns, and thus the dissipated energy, increases with this ratio. In cases where the DJL suggested a limiting amplitude caused by shear instabilities, ISWs accompanied by overturns were observed for *A_obs_/A_lim_* as small as 0.75 (Fig. 3). A possible explanation for the occurrence of ISW breaking below the limiting amplitude is that the wave breaking is initialized when the wave has an amplitude at, or above, the limiting amplitude, but continues even when the wave height is reduced in the breaking process. Alternatively the velocity perturbations due to surface waves could trigger the instability.

The probability of breaking does not only depend on *A_obs_*, but also on stratification. Therefore, the range of the limiting amplitudes determined for the seasonally changing stratifications from several years is large (3 m *≤ A_lim_ ≤*38 m) as well as the observed amplitude range of ISWs with *A_obs_/A_lim_* >1 (3.5 *m ≤ A_obs_ ≤*30 m). Thus, a small amplitude of an ISW or a strong stratification does not necessarily imply stability of the ISW. During all seasons, and for almost all stratifications, ISWs occurred in the field that had amplitudes exceeding the limiting amplitude for the relevant background stratification (Fig. 3A).

### Breaking mechanisms

The spatial evolution of a shear limited ISW was analysed in detail based on data from several stations deployed in the basin wide experiment (Fig. 1B). The background stratification was essentially the same at all measuring stations. Fig. 5 shows that the amplitude of the leading ISW (same wave as shown in Fig. 2) increased along the wave path until station B where it reached values above *A_lim_* (second square, *A_obs_/A_lim_*  = 1.17). Since wave energy increases with increasing wave amplitude as long as lake depth either remains constant or is also increasing, the ISW gained energy during the propagation from station A to B.

Thus between station A and B the ISW must have received energy from the steep-fronted basin-scale seiche. The increase in wave amplitude above the ratio *A_obs_/A_lim_* at station B suggests that the flux of energy from the basin scale front to the leading ISW was larger than the dissipation of energy by shear instabilities that might have set in between station A and B (Fig. 5, grey area). From station B onwards *A_obs_* declined and already at station C *A_obs_* was below *A_lim_* (*A_obs_/A_lim_*  = 0.96). Thus, during this time the shear instabilities transferred more energy from the ISW to turbulence than the ISW received from the front, causing a decline in wave amplitude. Note, that temperature inversions indicating wave breaking were observed at station C where the amplitude of the ISW was already below the limiting amplitude. These observations support the hypothesis that dissipation of energy by shear instabilities leads to a decline in wave amplitude and that wave breaking may continue even if the amplitude has declined to values below the limiting amplitude.

The amplitude of about one third of the waves occurring in Lake Überlingen was bounded by the breaking limit (Fig. 6, black columns). In 35% of these cases the amplitudes of the ISWs were larger than the breaking limit and these waves are thus predicted to carry a trapped core. During autumn, when the air temperature decreases and a mixed layer develops at the surface, the proportion of ISWs with amplitudes bounded by the breaking limit was smaller (Fig. 6). Both, breaking and shear limited waves can exceed their limiting amplitude by more than a factor of 1.5 (Fig. 3A), suggesting that the mechanism generating ISWs with amplitudes above the limiting amplitude is independent of the existence of a mixed surface layer. Thus, the probability of observing waves with a trapped core should only depend on the number of ISWs with amplitudes bounded by the breaking limit. However, about half (not one third) of the breaking ISWs exceeded the breaking limit at the measuring station (Fig. 6, grey columns). This suggests that breaking limited waves can hold their large amplitudes over a longer distance than shear limited waves, e.g. because they dissipate less of their energy on their way through the lake.

Although some of the breaking limited waves indeed were observed to either carry a core-like structure (Fig. 4A, C) or a perturbed trapped core (Fig. 4B), we did we did not observe trapped cores matching the idealized shapes simulated by the DJL equation or dynamical models [11], [14]. This suggests that the core structure of the observed waves is highly unstable and probably does not support transport over large distances.

Dynamical simulations were performed to determine the extent to which trapped cores are robust to perturbations in the background stratification. Given the range of stratifications throughout the seasonal cycle, the question of which aspects of the stratification profile were absolutely necessary for trapped cores to form was first considered. The exponential stratification profile [11] was found to both successfully represent features of the field data (e.g. by changing the top to bottom density difference the long wave speed could be matched to the measured value) and yield robust trapped cores in time dependent simulations, and was thus adopted for all simulations shown. The simulations were performed with a second order, finite volume code, with code details and typical resolution tests discussed in Stastna and Lamb [22]. Trapped cores formed spontaneously from DJL initial conditions using a variety of parameterizations for fluid properties in the core region (the DJL equation cannot uniquely specify these [14]). The trapped cores were quasi-steady and persisted for long times (in analogy to trapped cores in trapped waves discussed by Soontiens et al. [23]). Following the suggestions made in Lamb and Farmer [21], the trapped cores were subsequently subjected to a variety of upstream introduced perturbations (e.g. patches of light fluid in the top 1–2% of the water column). Figure 7A shows a quasi-steady core, while Figure 7B–D shows the nearly complete disintegration of the core after being subjected to small disturbances in the water column upstream of the wave. The core is profoundly altered 40 minutes after perturbations are introduced, and 60 minutes later (Fig. 7D) the core is nearly completely destroyed. Despite of this, the main wave continues to propagate largely unhindered. These simulations indicate that the stability of the cores, but not of the waves in general, is highly sensitive to perturbations of the near-surface water, suggesting that whereas ISWs with amplitudes sufficiently large for the formation of a trapped core are observable under field conditions, cores with shapes matching DJL or idealized dynamical simulations are comparatively rare. It also implies that Lagrangian transport by such waves will be greatly reduced from theoretical predictions based on trapped cores.

### Conclusions

Our observations suggest that ISWs in the field break in the open water far from the bottom boundaries when their amplitudes are larger than the limiting amplitudes derived via the DJL equation. Breaking ISWs can sometimes develop amplitudes more than 1.5 times larger than their limiting amplitude probably due to the continuing energy flux from the basin-scale seiche. A statistical comparison of the ISWs with different breaking mechanisms suggests that breaking limited waves are more likely to preserve large amplitudes, whereas shear instabilities result in a decline of the ISW amplitude. These findings have several implications:

At least one half of the breaking ISWs broke due to shear instabilities. Observations of density inversions combined with large values of *A_obs_/A_lim_* suggest that shear limited waves dissipate energy and we can speculate that they might do this even while receiving energy from the steepened seiche. In order to judge the contribution of shear limited waves to the energy cascade, it is thus necessary to monitor and quantify the dissipation rates together with the wave amplitudes at several measuring stations within the lake.

A substantial number of the waves with overturns were breaking limited waves. Convectively breaking waves are assumed to generate trapped cores that are potentially capable to cause transport over large distances [11]. However, although we found indications of convective instabilities in most of the breaking limited ISWs, we did not observe trapped cores matching the idealized shapes predicted by theory in any of the breaking limited ISWs. Trapped core formation and structure might be affected by surface waves [24] and seems to be highly sensitive to small instabilities in the core itself [14] and to small perturbations of the near-surface water, as suggested by our dynamical simulations. Lien et al. [25] recently presented the first detailed observation of a trapped core wave of depression, showing that the core continuously exchanged water with its surroundings. Hence, it seems rather unlikely that trapped cores in ISWs of depression can be stable in natural environments and the lack of ideal shaped trapped cores in our observations may be typical for lakes or the ocean. The ecological impact of breaking limited waves remains to be analyzed under these conditions, i.e. how the instability of the core affects the transport abilities of these waves and whether mixing becomes important in this context. It is interesting to note that many of the perturbations that may lead to the breakdown of the trapped cores would be absent near the bottom.

Internal tides can generate ISWs in the ocean in a similar way as the surges in lakes and might result in the generation of ISWs exceeding their limiting amplitude. Shear unstable waves in deep ocean water were e.g. observed by Moum et al. [13]. While it is reasonable to assume that the limiting amplitudes of ISWs in the ocean can be equally well described via the DJL equation as in Lake Überlingen, current velocities could have a larger effect in the ocean, influencing both, the limiting amplitude and the breaking mechanism [10].
